# Impact of Icosapent Ethyl on Cardiovascular Risk Reduction in Patients With Heart Failure in REDUCE‐IT

**DOI:** 10.1161/JAHA.121.024999

**Published:** 2022-04-04

**Authors:** Senthil Selvaraj, Deepak L. Bhatt, Ph. Gabriel Steg, Michael Miller, Eliot A. Brinton, Terry A. Jacobson, Rebecca A. Juliano, Lixia Jiao, Jean‐Claude Tardif, Christie M. Ballantyne, the REDUCE‐IT Investigators

**Affiliations:** ^1^ Division of Cardiology Department of Medicine Hospital of the University of Pennsylvania Philadelphia PA; ^2^ Brigham and Women’s Hospital Heart & Vascular Center Harvard Medical School Boston MA; ^3^ Université Paris‐Cité AP‐HP (Assistance Publique‐Hôpitaux de Paris) Hôpital Bichat FACT (French Alliance for Cardiovascular Trials) INSERM U‐1148 Paris France; ^4^ Department of Medicine University of Maryland School of Medicine Baltimore MD; ^5^ Utah Lipid Center Salt Lake City UT; ^6^ Office of Health Promotion and Disease Prevention Department of Medicine Emory University School of Medicine Atlanta GA; ^7^ Amarin Pharma, Inc (Amarin) Bridgewater NJ; ^8^ Montreal Heart Institute Université de Montréal Montreal QC Canada; ^9^ Department of Medicine Baylor College of Medicine Center for Cardiovascular Disease Prevention Methodist DeBakey Heart and Vascular Center Houston TX

**Keywords:** eicosapentaenoic acid, heart failure, prevention, Heart Failure

## Abstract

**Background:**

Patients with heart failure (HF) are at high risk for atherosclerotic cardiovascular disease. Studies of atherothrombotic treatments in this population have been disappointing to date. Icosapent ethyl reduced the risk of atherosclerotic cardiovascular disease among a broad array of statin‐treated patients at elevated risk for atherosclerotic cardiovascular disease. Whether the treatment benefits of icosapent ethyl are consistent among those with HF is unknown.

**Methods and Results:**

REDUCE‐IT (Reduction of Cardiovascular Events With Icosapent Ethyl–Intervention Trial) randomized 8179 participants, including 1446 (17.7%) patients with a history of HF (icosapent ethyl, N=703; and placebo, N=743). The primary end point was a composite of cardiovascular death, nonfatal myocardial infarction, nonfatal stroke, coronary revascularization, or unstable angina. We used Cox regression to estimate the risk of outcomes of participants with and without HF. We estimated the placebo‐controlled change in triglycerides and hs‐CRP (high‐sensitivity C‐reactive protein) from baseline to 2 years. Among 1446 patients with HF, median age was 63.0 years, median body mass index was 31.0 kg/m^2^, and more were men (69.3%). Icosapent ethyl reduced triglycerides (median reduction, 33.5 mg/dL, or 15.4%; *P*<0.0001) and hs‐CRP (35.1%; *P*<0.0001) compared with placebo, similar to patients without HF (*P*‐interaction>0.90). The treatment effect on the primary end point in patients with HF history (hazard ratio [HR], 0.87; 95% CI, 0.70–1.08) was consistent with the effects observed in patients without HF history (HR, 0.73; 95% CI, 0.65–0.81) (*P*‐interaction=0.13).

**Conclusions:**

In REDUCE‐IT, icosapent ethyl provided similar improvements in triglyceride levels and hs‐CRP as well as similar cardiovascular risk reduction in patients with and without HF.

**Registration:**

URL: https://www.clinicaltrials.gov; Unique identifier: NCT01492361.

Patients with heart failure (HF) are at high risk for atherosclerotic cardiovascular disease (ASCVD),^1^, ^2^ as designated by professional society guidelines.^3^ Trials of low‐dose anticoagulation,^4^ PCSK9 (proprotein convertase subtilisin/kexin type 9) inhibitors,^5^ as well as statins^2^, ^6^ in patients with HF have been disappointing to date, and guideline recommendations for statins among patients with HF remain weak.^3^ However, low‐dose, mixed omega‐3 treatment marginally reduced cardiovascular events among patients with HF in a trial enrolled in a single country.^7^ In the international REDUCE‐IT (Reduction of Cardiovascular Events With Icosapent Ethyl–Intervention Trial),^8^ treatment with icosapent ethyl significantly reduced ischemic events among statin‐treated patients with controlled low‐density lipoprotein cholesterol, elevated triglyceride levels, and increased ASCVD risk.^9^ Whether these benefits are consistent between patients with and without HF has not been explored.

The data that support the findings of this study may be made available from the corresponding author on reasonable request. REDUCE‐IT randomized 8179 participants, including 1446 (17.7%) patients with a history of HF (New York Heart Association class I–III) who were assigned to icosapent ethyl (N=703) or placebo (N=743). Verbal and written informed consent were obtained from all study participants, and all sites were approved by institutional review boards. HF was based on reports from a cardiovascular history case report form. The primary end point was a composite of cardiovascular death, nonfatal myocardial infarction, nonfatal stroke, coronary revascularization, or unstable angina. The key secondary end point was a composite of cardiovascular death, nonfatal myocardial infarction, or nonfatal stroke. We also evaluated the risk for HF hospitalization by history of prevalent HF at baseline. HF hospitalization was defined by all of the following: (1) admission with a primary diagnosis of HF; (2) admission for at least 24 hours; (3) new or worsening symptoms attributable to HF on presentation; (4) objective evidence of new or worsening HF (as evidenced by a combination of physical examination and laboratory criteria); and (5) initiation or intensification of treatment specifically for HF.

In this post hoc analysis, we estimated hazard ratios (HRs) and 95% CIs among patients with and without history of HF using Cox proportional‐hazards models that included trial‐group assignment as a covariate, stratified according to cardiovascular risk category (established cardiovascular disease or diabetes with risk factors), geographic region, and baseline use of ezetimibe. “Log‐log plots” and analysis of Schoenfeld residuals were used to support the proportionality of hazards assumption. We assessed the interaction of HF and treatment with several prespecified outcomes.

Among 1446 patients with HF, median age was 63.0 years, median body mass index was 31.0 kg/m^2^, and more were men (69.3%), similar to patients without HF. However, patients with HF were more likely to be in the cardiovascular disease stratum (84.0% versus 67.9%), be White race (95.0% versus 89.2%), have hypertension (93.7% versus 85.1%), have kidney impairment (27.0% versus 21.2%), have a history of atrial fibrillation (16.3% versus 7.7%), have combined dyslipidemia (triglycerides ≥200 mg/dL plus high‐density lipoprotein cholesterol ≤35 mg/dL; 22.9% versus 19.1%), and have higher median hs‐CRP (high‐sensitivity C‐reactive protein) (2.6 mg/L versus 2.1 mg/L) (*P*<0.05 for all comparisons). They were less likely to have diabetes (53.9% versus 58.7%), use ezetimibe (2.9% versus 7.2%), be from a Westernized country (33.0% versus 79.2%), and had lower median baseline eicosapentaenoic acid levels (21.6 μg/mL versus 27.0 μg/mL) (*P*<0.05 for all comparisons). Patients with HF who had a history of atrial fibrillation were anticoagulated at similar rates to patients without HF who had a history of atrial fibrillation (63.1% versus 62.1%), although direct oral anticoagulant use was low in both groups (9.3% and 13.0%, respectively, of patients with a history of atrial fibrillation). Patients with HF had median baseline low‐density lipoprotein cholesterol of 76.0 mg/dL, high‐density lipoprotein cholesterol of 39.0 mg/dL, and triglycerides of 217.5 mg/dL, similar to patients without HF.

Among patients with HF, icosapent ethyl reduced triglycerides (median reduction, 33.5 mg/dL, or 15.4% [95% CI, 10.7%–20.1%; *P*<0.0001]) and hs‐CRP (35.1%; 95% CI, 25.2%–45.3%; *P*<0.0001) from baseline to 2 years compared with placebo. These reductions were similar to patients without HF (*P*‐interaction=0.96 for triglycerides, and *P*=0.98 for hs‐CRP). A primary event occurred in 160 of 703 (22.8%) icosapent ethyl–treated patients compared with 187 of 743 (25.2%) placebo‐treated patients (Figure 1). The treatment effect on the primary end point in patients with HF history (HR, 0.87; 95% CI, 0.70–1.08) was consistent with the effects observed in patients without HF history (HR, 0.73; 95% CI, 0.65–0.81) (*P*‐interaction=0.13). Similar findings were observed for the key secondary composite end point (HF history: HR, 0.85; 95% CI, 0.66–1.08; no HF history: HR, 0.71; 95% CI, 0.62–0.81) (*P*‐interaction=0.21) and for components of the primary end point. Furthermore, as reported previously, the risk for HF hospitalization was not reduced by treatment with icosapent ethyl,^9^ and this risk was not significantly different by history of prevalent HF at baseline (*P*‐interaction=0.25) (Figure 2).

**Figure 1:**
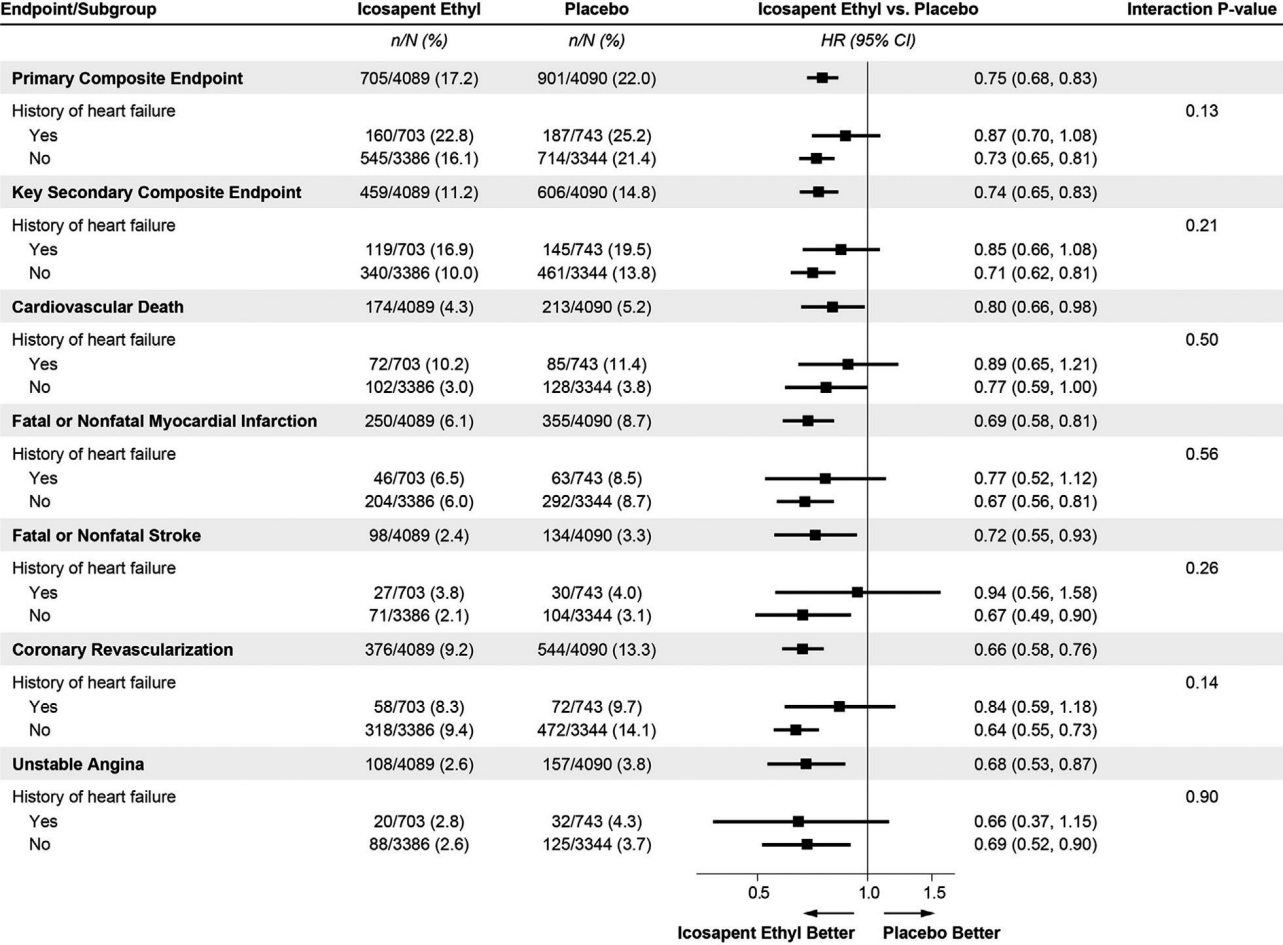
Treatment effect of icosapent ethyl compared with placebo in patients with and without heart failure across efficacy end points. Icosapent ethyl appears similarly efficacious in patients with and without history of heart failure across all displayed end points.

**Figure 2:**
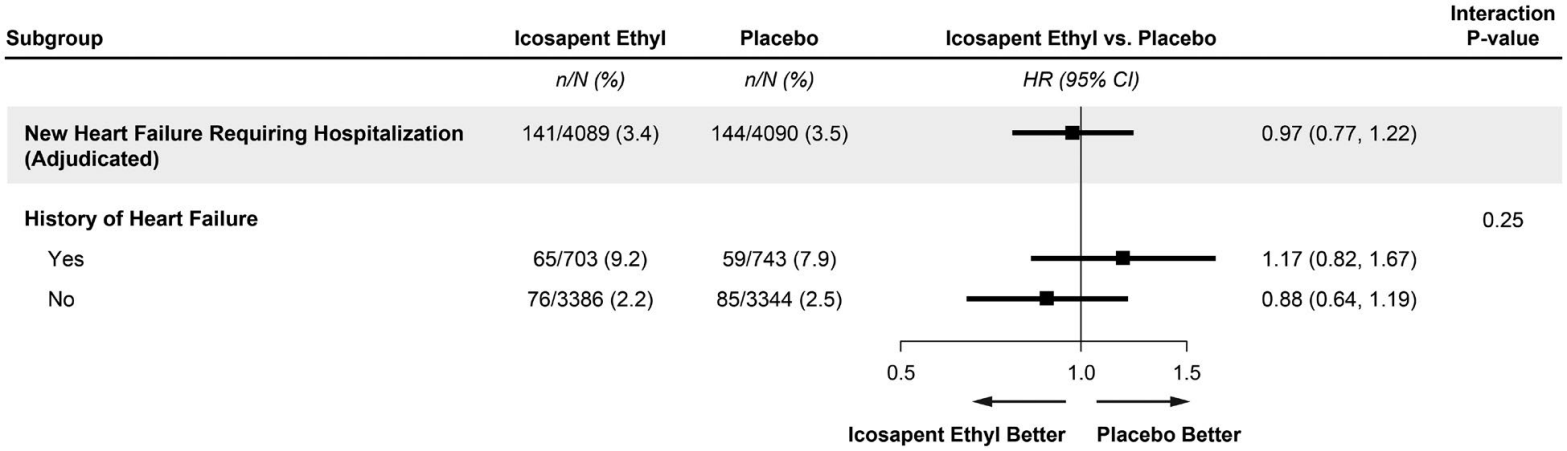
Risk for heart failure requiring hospitalization by treatment assignment in patients with and without prevalent heart failure. Icosapent ethyl did not reduce the risk for heart failure hospitalization compared with placebo, and this was not significantly different by history of prevalent heart failure. HR indicates hazard ratio.

In patients with HF receiving statin treatment, our findings suggest that icosapent ethyl is similarly efficacious in reducing the risk of ASCVD irrespective of history of HF. The reductions observed in triglycerides as well as hs‐CRP are notable, particularly given recent interest in pharmacotherapies to reduce these biomarkers in patients with HF.^10^, ^11^ We have previously reported that icosapent ethyl did not reduce new‐onset HF (4.1%) compared with placebo (4.3%) (HR, 0.95; 95% CI, 0.77–1.17).^9^ New HF requiring hospitalization was likewise similar between icosapent ethyl and placebo (3.4% versus 3.5%; HR, 0.97; 95% CI, 0.77–1.22), and we now report that this treatment effect was not significantly different by history of prevalent HF. Although icosapent ethyl did not reduce new‐onset HF compared with placebo, the similar reductions in ASCVD in patients with and without HF are clearly still of clinical relevance.^1^, ^2^ Indeed, the prevention of ASCVD in patients with HF represents an additional therapeutic pathway to improving morbidity in a high‐risk population who may be particularly vulnerable to the effects of atherosclerosis.^1^

Limitations of this analysis include lack of data on ejection fraction to determine consistency of benefit when applied to patients with HF with reduced or preserved ejection fraction as well as information on HF cause. Furthermore, history of HF at baseline was based on report and not verified by additional criteria. Finally, interaction analyses between subgroups may be underpowered to detect significant treatment effect differences between groups.^12^ In this way, the treatment effect estimate was qualitatively stronger in those without HF than those with HF, although the point estimate in those with HF still favored icosapent ethyl treatment. Strengths include randomization of nearly 1500 patients with HF, median follow‐up duration of ≈4.6 years, and collection of relevant biomarkers.

In REDUCE‐IT, icosapent ethyl provided similar improvements in triglyceride levels and hs‐CRP as well as similar cardiovascular risk reduction in patients with and without HF. These data suggest consistency of benefit from icosapent ethyl treatment in patients with or without HF.

## Sources of Funding

REDUCE‐IT (Reduction of Cardiovascular Events With Icosapent Ethyl–Intervention Trial) was sponsored by Amarin Pharma.

## Disclosures

Dr Selvaraj receives research support from the Doris Duke Charitable Foundation (Physician Scientist Fellowship Award 2020061), the Measey Foundation, Institute for Translational Medicine and Therapeutics (Junior Investigator Preliminary/Feasibility Grant Program award and Translational Bio‐Imaging Center award), and the American Society of Nuclear Cardiology (Institute for the Advancement of Nuclear Cardiology award). Dr Bhatt serves as the Chair and International Principal Investigator for REDUCE‐IT (Reduction of Cardiovascular Events With Icosapent Ethyl–Intervention Trial), with research funding from Amarin to Brigham and Women’s Hospital. Dr Bhatt also discloses the following relationships: Advisory Board: Bayer, Boehringer Ingelheim, Cardax, CellProthera, Cereno Scientific, Elsevier Practice Update Cardiology, Janssen, Level Ex, Medscape Cardiology, Merck, MyoKardia, NirvaMed, Novo Nordisk, PhaseBio, PLx Pharma, Regado Biosciences, and Stasys; Board of Directors: Boston VA Research Institute, DRS.LINQ (stock options), Society of Cardiovascular Patient Care, and TobeSoft; Chair: Inaugural Chair, American Heart Association Quality Oversight Committee; Data Monitoring Committees: Acesion Pharma, Assistance Publique–Hôpitaux de Paris, Baim Institute for Clinical Research (formerly Harvard Clinical Research Institute, for the PORTICO trial, funded by St. Jude Medical, now Abbott), Boston Scientific (Chair, PEITHO trial), Cleveland Clinic (including for the ExCEED trial, funded by Edwards), Contego Medical (Chair, PERFORMANCE 2), Duke Clinical Research Institute, Mayo Clinic, Mount Sinai School of Medicine (for the ENVISAGE trial, funded by Daiichi Sankyo; for the ABILITY‐DM trial, funded by Concept Medical), Novartis, Population Health Research Institute; Rutgers University (for the National Institutes of Health–funded MINT Trial); Honoraria: American College of Cardiology (ACC) (Senior Associate Editor, *Clinical Trials and News*, ACC.org; Chair, ACC Accreditation Oversight Committee), Arnold and Porter law firm (work related to Sanofi/Bristol‐Myers Squibb clopidogrel litigation), Baim Institute for Clinical Research (formerly Harvard Clinical Research Institute; RE‐DUAL PCI clinical trial steering committee funded by Boehringer Ingelheim; AEGIS‐II executive committee funded by CSL Behring), Belvoir Publications (Editor in Chief, *Harvard Heart Letter*), Canadian Medical and Surgical Knowledge Translation Research Group (clinical trial steering committees), Cowen and Company, Duke Clinical Research Institute (clinical trial steering committees, including for the PRONOUNCE trial, funded by Ferring Pharmaceuticals), HMP Global (Editor in Chief, *Journal of Invasive Cardiology*), *Journal of the American College of Cardiology* (Guest Editor; Associate Editor), K2P (Co‐Chair, interdisciplinary curriculum), Level Ex, Medtelligence/ReachMD (continuing medical education [CME] steering committees), MJH Life Sciences, Piper Sandler, Population Health Research Institute (for the COMPASS operations committee, publications committee, steering committee, and US national coleader, funded by Bayer), Slack Publications (Chief Medical Editor, *Cardiology Today’s Intervention*), Society of Cardiovascular Patient Care (Secretary/Treasurer), WebMD (CME steering committees), Wiley (steering committee); Other: *Clinical Cardiology* (Deputy Editor), NCDR‐ACTION Registry Steering Committee (Chair), VA CART Research and Publications Committee (Chair); Research Funding: Abbott, Afimmune, Aker Biomarine, Amarin, Amgen, AstraZeneca, Bayer, Beren, Boehringer Ingelheim, Boston Scientific, Bristol‐Myers Squibb, Cardax, CellProthera, Cereno Scientific, Chiesi, CSL Behring, Eisai, Ethicon, Faraday Pharmaceuticals, Ferring Pharmaceuticals, Forest Laboratories, Fractyl, Garmin, HLS Therapeutics, Idorsia, Ironwood, Ischemix, Janssen, Javelin, Lexicon, Lilly, Medtronic, Merck, Moderna, MyoKardia, NirvaMed, Novartis, Novo Nordisk, Owkin, Pfizer, PhaseBio, PLx Pharma, Recardio, Regeneron, Reid Hoffman Foundation, Roche, Sanofi, Stasys, Synaptic, The Medicines Company, and 89Bio; Royalties: Elsevier (Editor, *Braunwald’s Heart Disease*); Site Co‐Investigator: Abbott, Biotronik, Boston Scientific, CSI, St. Jude Medical (now Abbott), Philips, and Svelte; Trustee: American College of Cardiology; Unfunded Research: FlowCo and Takeda. Dr Steg has received grant support and fees for serving on a steering committee from Amarin, grant support and fees for serving on a steering committee from Bayer/Janssen, grant support and lecture fees from Merck, grant support, fees for serving as cochair of the ODYSSEY outcomes trial and as cochair of the SCORED trial, consulting fees, and lecture fees from Sanofi, consulting fees and lecture fees from Amgen, consulting fees, lecture fees, and fees for serving on an event committee from Bristol‐Myers Squibb, fees for serving on an executive steering committee from Boehringer Ingelheim, fees for serving on an event committee from Pfizer, consulting fees and fees for serving on an executive steering committee from Novartis, consulting fees from Regeneron and Lilly, consulting fees and fees for serving as cochair of the THEMIS trial from AstraZeneca, and grant support and fees for serving as chair of the data and safety monitoring committee for the ATPCI trial and as chair of the CLARIFY registry from Servier. Dr Miller has received consulting fees from Amarin and Akcea. Dr Brinton has received lecture fees from Boehringer, Janssen, Kaneka, and Novo Nordisk, consulting fees and lecture fees from Amarin, Amgen, AstraZeneca, Kowa, and Regeneron, and consulting fees from 89 Bio, Dalcor, Esperion, and Medicure. Dr Jacobson has received consulting fees from AstraZeneca, Amgen, Novartis, Esperion, and Regeneron/Sanofi. Dr Juliano and Dr Jiao are employed by and are stock shareholders of Amarin Pharma. Dr Tardif has received grant support from AstraZeneca, Esperion, and Ionis, grant support and consulting fees from DalCor, grant support and fees for serving as cochairman of an executive committee from Pfizer, grant support and fees for serving on an executive committee from Sanofi, and grant support and consulting fees from Servier and holding a minor equity interest in DalCor and a patent (US 9 909 178 B2) on dalcetrapib for therapeutic use. Dr Ballantyne has received consulting fees from AstraZeneca, Eli Lilly, Matinas BioPharma, Merck, Boehringer Ingelheim, Novo Nordisk, Denka Seiken, and Gilead and grant support (paid to his institution) and consulting fees from Amarin, Amgen, Esperion, Novartis, Regeneron, Sanofi‐Synthelabo, and Akcea.

## Supporting information

REDUCE‐IT Trial Investigators
